# Developmental Changes in Sleep Oscillations during Early Childhood

**DOI:** 10.1155/2017/6160959

**Published:** 2017-08-15

**Authors:** Eckehard Olbrich, Thomas Rusterholz, Monique K. LeBourgeois, Peter Achermann

**Affiliations:** ^1^Max Planck Institute for Mathematics in the Sciences, Leipzig, Germany; ^2^Institute of Pharmacology and Toxicology, University of Zurich, Zurich, Switzerland; ^3^University Hospital of Child and Adolescent Psychiatry and Psychotherapy, University of Bern, Bern, Switzerland; ^4^Sleep and Development Laboratory, Department of Integrative Physiology, University of Colorado Boulder, Boulder, CO, USA; ^5^The KEY Institute for Brain-Mind Research, Department of Psychiatry, Psychotherapy and Psychosomatics, University Hospital of Psychiatry, Zurich, Switzerland; ^6^Zurich Center for Interdisciplinary Sleep Research, University of Zurich, Zurich, Switzerland; ^7^Neuroscience Center Zurich, University and ETH Zurich, Zurich, Switzerland

## Abstract

Although quantitative analysis of the sleep electroencephalogram (EEG) has uncovered important aspects of brain activity during sleep in adolescents and adults, similar findings from preschool-age children remain scarce. This study utilized our time-frequency method to examine sleep oscillations as characteristic features of human sleep EEG. Data were collected from a longitudinal sample of young children (*n* = 8; 3 males) at ages 2, 3, and 5 years. Following sleep stage scoring, we detected and characterized oscillatory events across age and examined how their features corresponded to spectral changes in the sleep EEG. Results indicated a developmental decrease in the incidence of delta and theta oscillations. Spindle oscillations, however, were almost absent at 2 years but pronounced at 5 years. All oscillatory event changes were stronger during light sleep than slow-wave sleep. Large interindividual differences in sleep oscillations and their characteristics (e.g., “ultrafast” spindle-like oscillations, theta oscillation incidence/frequency) also existed. Changes in delta and spindle oscillations across early childhood may indicate early maturation of the thalamocortical system. Our analytic approach holds promise for revealing novel types of sleep oscillatory events that are specific to periods of rapid normal development across the lifespan and during other times of aberrant changes in neurobehavioral function.

## 1. Introduction

The electroencephalogram (EEG) is a fundamental method for identifying sleep/wakefulness states, quantifying sleep-related cortical activity and assessing sleep regulation. Slow oscillations, delta activity, and sleep spindles are prominent features of the sleep EEG during nonrapid eye movement (NREM) sleep. Visual detection of some features of the sleep EEG (e.g., sleep spindles) can be challenging because such events may be masked by high-amplitude slow waves. This is true during early childhood, a period of rapid maturation of brain activity during sleep and a time when the amplitude of slow waves is relatively high [1]. Quantitative EEG analysis is a strong approach to amending this challenge.

The sleep EEG can be analyzed in several ways: (i) the time domain (e.g., period-amplitude analysis), which provides information about the incidence and amplitude of waves [2, 3]; (ii) the frequency domain (e.g., spectral analysis), which decomposes signals into constituting frequency components; or (iii) time-frequency analysis methods (e.g., spectrograms, wavelet analysis, and matching pursuit), which combine elements of both domains [4]. In this paper, we use a time-frequency approach based on adaptive autoregressive models that allows detection of oscillatory events without requiring the definition of prespecified frequency bands (i.e., known oscillations such as slow waves or sleep spindles) and thus is more sensitive to interindividual differences in the frequencies of oscillatory activity. Such approaches may also be best suited for uncovering oscillatory events that are unique to periods of rapid early neurodevelopmental change, which show high variability and commonly do not unfold linearly but in series of stepwise spurts and plateaus (e.g., [5, 6]).

The benefits of sleep for brain function and learning as reflected in plastic changes across the cortex during development are increasingly recognized (e.g., [7]). Oscillatory events during sleep are proposed to be biomarkers of neurodevelopmental brain plasticity. In particular, slow waves, delta activity, and spindles generated by thalamocortical networks [8, 9] might reflect developmental changes in functional brain connectivity within the same physiological structures [10, 11]. To date, reports of interindividual variability and longitudinal development of sleep oscillations in early life remain scarce.

Here, we utilized our previously published time-frequency analysis method based on autoregressive modelling of short overlapping EEG segments [12–14] to characterize transient oscillatory activity in the longitudinal nighttime sleep EEG recordings of children at ages 2, 3, and 5 years. In addition to average developmental changes, our analytic approach allowed for sensitive assessment of interindividual variation in our measures. Furthermore, we examined how specific oscillatory events herald the onset of sleep (i.e., the transition from waking to sleep).

## 2. Methods

### 2.1. Subjects and Protocol

Eight healthy children (3 males; 6 Caucasians) were studied longitudinally at three ages [2.8 ± 0.2 (SEM; 2Y); 3.8 ± 0.2 (3Y); 5.9 ± 0.2 (5Y) years]. Recruitment, screening, and inclusion/exclusion criteria are detailed in our previous publications [10, 15].

During the 5 days before an overnight sleep recording, children followed a sleep stabilization schedule. They slept in their habitual environment (i.e., home, day care, and family care) with a minimal sleep opportunity of 12.5 h (2Y and 3Y) or 12 h (5Y) per 24 h day. The schedule included a nap opportunity of at least 45 min at 2Y for all children and at 3Y for seven of the children (one child had stopped napping). The stabilization phase served to minimize sleep restriction and to entrain the circadian system. Compliance with the prescribed schedule was verified with sleep diaries, daily parent reports, and actigraphic recordings. Caffeine consumption and medication affecting sleep, alertness, or the circadian system were prohibited during the entire study.

Overnight in-home sleep EEG was obtained after 13 hours of prior wakefulness [no napping; bedtime 19 : 58 ± 0 : 11 (SEM) at 2Y, 20 : 05 ± 0 : 09 at 3Y, 19 : 59 ± 0 : 13 at 5Y; wake time 6 : 47 ± 0 : 11 at 2Y, 6 : 52 ± 0 : 14 at 3Y, 6 : 34 ± 0 : 14 at 5Y] using a portable 16-channel EEG recorder (Vitaport 3, Temec Instruments, Kerkrade, The Netherlands). Four EEG derivations (C3A2, C4A1, O1A2, and O2A1; standard 10–20 system), EMG, and EOG were recorded. Data were sampled at 128 Hz (EEG filter 0.16–30 Hz; 2Y) and at 256 Hz (0.16–70 Hz; 3Y and 5Y). Sleep stages were visually scored for 30 s epochs (C3A2) according to standard criteria [16] by a reliable scorer (MKL).

### 2.2. Spectral Analysis

Power density spectra (derivation C3A2) were calculated for consecutive 30 s epochs [FFT, Tukey window (tapered cosine, ratio of cosine tapered section length to the entire window length 0.5), average of ten 4 s epochs overlapping by 1 s; matched with sleep stages; VitaScore, Temec Instruments, Kerkrade, The Netherlands] resulting in a frequency resolution of 0.25 Hz. Epochs were excluded whenever power in the 20–40 Hz and 0.75–4.5 Hz band exceeded a threshold based on a moving average determined over twenty 30 s epochs.

### 2.3. Detection and Analysis of Oscillatory Events

Oscillatory events in the sleep EEG were identified in derivation C3A2 by applying an algorithm published previously [13]. Data recorded with 256 Hz (3Y and 5Y) were resampled at 128 Hz. Basically, the EEG was modelled as a superposition of maximal 4 stochastically driven harmonic oscillators with their damping and frequency varying in time. This was achieved by fitting an autoregressive model of order 8 [AR(8)-model] to overlapping 1 s segments of the EEG time series using the sampling interval (1/128 s) as step size. In previous applications, a step size of 8 samples was applied. Oscillatory events were detected whenever the damping at one or more frequencies was smaller than a predefined threshold (*γ* ≤ 6.6 s^−1^; [13]). If 1 s segments corresponding to distinct events were overlapping in time and frequency, they were merged into a single event. For a detailed description of the original algorithm and its application to adult human sleep EEG data, see Olbrich and Achermann [13] and Olbrich et al. [14, 17]. In the present paper, events were characterized by their time of occurrence, mean frequency, and duration.

**Figure 1:**
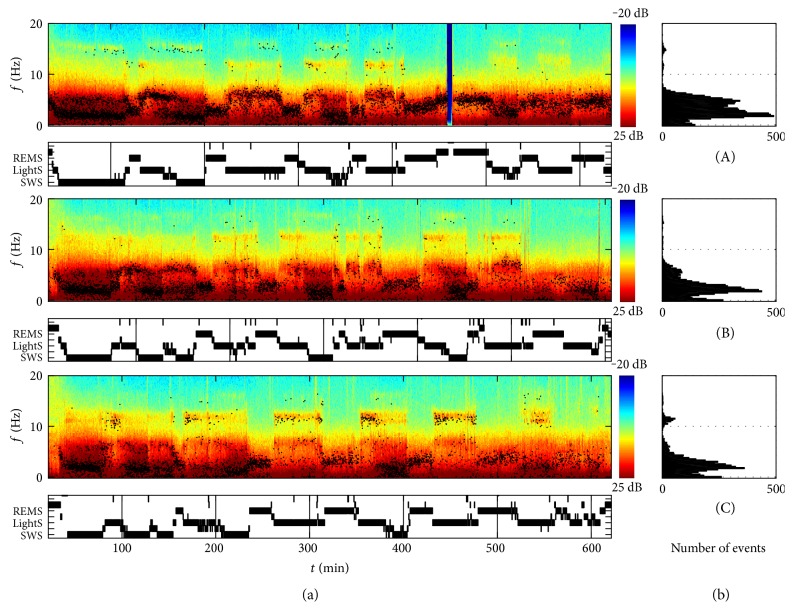
Spectrograms, oscillatory events (derivation C3A2), and hypnograms of a boy (same child shown in Supplementary Figure S1) recorded longitudinally at ages 2 years (A), 3 years (B), and 5 years (C). For each recording, the following data are illustrated. (a)—top: spectrogram and superimposed oscillatory events (black dots). Spectra are color coded on a logarithmic scale (0 dB = 1 *μ*V^2^/Hz). (b) Frequency distribution of oscillatory events. (a)—bottom: hypnogram (REMS: REM sleep; LightS: NREM sleep stages 1 and 2; SWS: NREM sleep stages 3 and 4). The dark blue bar reflects a short disconnection of the child.

**Figure 2:**
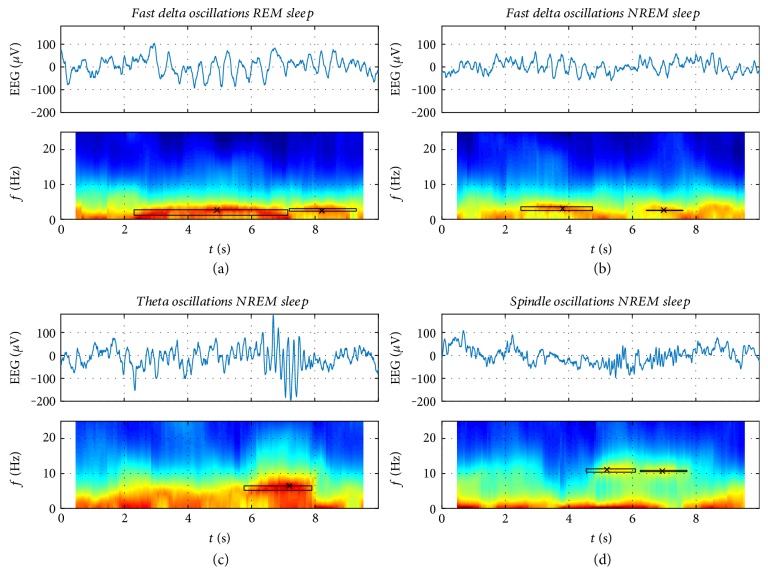
Illustration of single events. For each event, the following data are illustrated: 10 s epoch of EEG data (derivation C3A2) and spectrogram with the detected events marked by a rectangle. The length of the horizontal rectangle indicates the duration of the event and its height the minimal and maximal frequency. The cross indicates the frequency *f*_events_ at the time of minimal damping. Color-coded spectrogram estimated using AR(8) models on overlapping 1 s segments (warmer colors denote higher power). Spectrograms start 0.5 s later and ends 0.5 s earlier due to the fact that a 1 s segment was moved through the data. All data are from children at 3 years. (a) Fast delta oscillations in REM sleep (same child shown in Supplementary Figure S2); (b) fast delta oscillations in NREM sleep stage 2 (same child shown in Supplementary Figure S2); (c) theta oscillations in NREM sleep stage 2 (same child shown in Supplementary Figure S4); and (d) sleep spindles in NREM sleep stage 2 (same child shown in Supplementary Figure S6) are illustrated.

Detected events in the sleep EEG of one child superimposed on the spectrogram are illustrated in Figure 1(a) for the three longitudinal recordings (data of all eight children are provided in Supplementary Figures S1–S8 available online at https://doi.org/10.1155/2017/6160959, top panels). In Figure 2, 10 s epochs of sleep EEG (top) and examples of detected events (rectangles) are illustrated in combination with color-coded spectrograms (bottom) estimated using AR(8) models on overlapping 1 s segments. Previously, events were characterized by the frequency *f*_events_ at the time of minimal damping [13]. Figure 2 shows this frequency (denoted by “*x*”) together with the minimal and maximal frequency (height of rectangle), and the length of the rectangle indicates the duration of the event.

**Figure 3:**
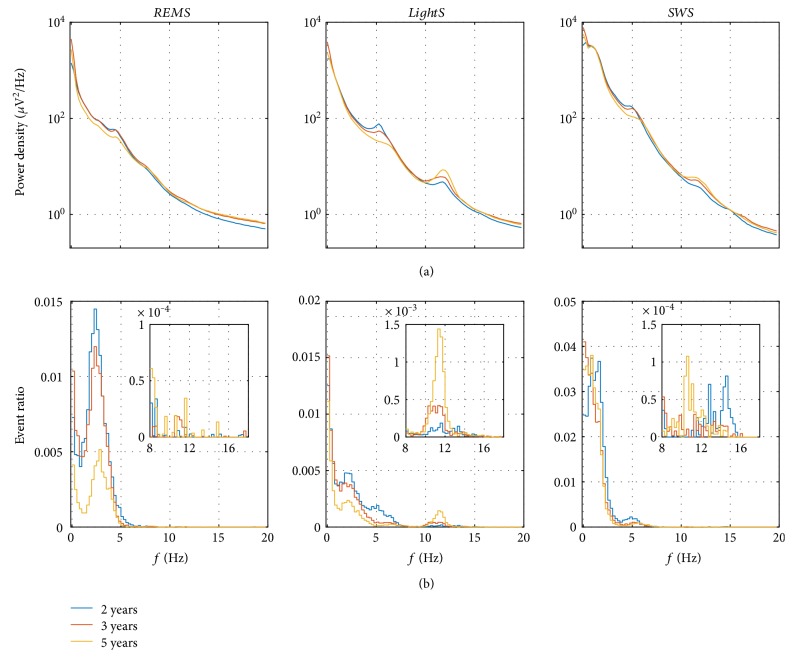
(a) Mean (*n* = 8) power density spectra (geometric mean) and (b) event ratios of REM sleep (REMS), light sleep (LightS, NREM sleep stages 1 and 2), and slow-wave sleep (SWS, NREM sleep stages 3 and 4) recorded longitudinally at 2 (blue), 3 (red), and 5 years (orange) of age. The insets in the event ratios are a zoom into the 8–18 Hz range, and the frequency axis of inset and main plot do correspond (e.g., 10 Hz is at the same location in both plots). For a statistical assessment of the longitudinal changes in specific frequency bands, see Figure 4.

**Figure 4:**
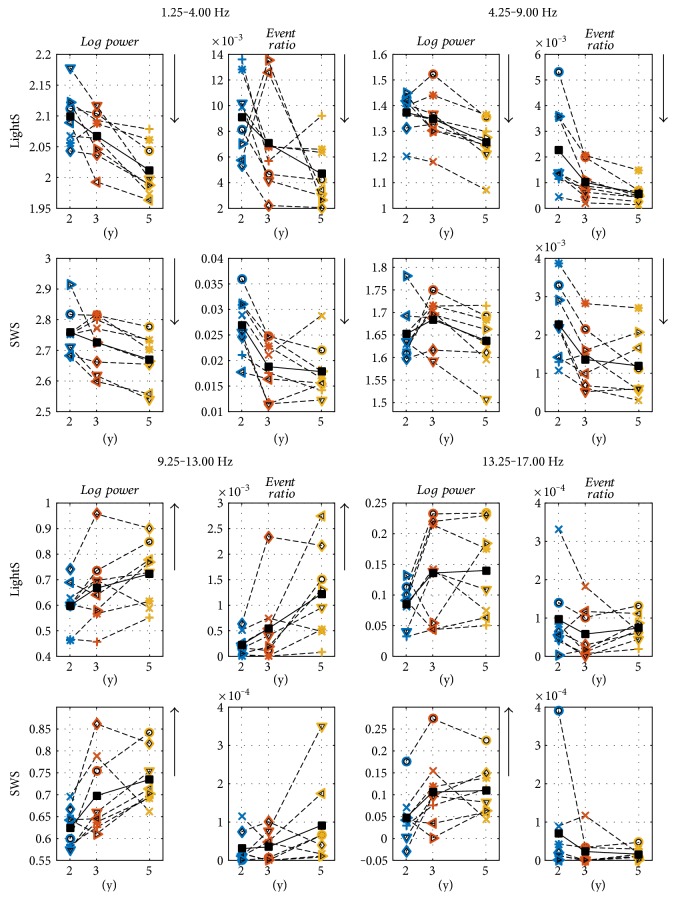
Mean power (log10) and event ratios of three frequency bands (delta 1.25–4.0 Hz; theta 4.25–8 Hz; and sigma 9.25–13.0 and 13.25–17.0 Hz) at three longitudinal time points (2, 3, and 5 years of age) to illustrate the developmental change in NREM sleep. Light sleep (LightS: NREM sleep stages 1 and 2) and slow-wave sleep (SWS: NREM sleep stages 3 and 4) were analyzed. Dashed lines and the different symbols illustrate individual children and the black squares and lines average data (*n* = 8). Arrows indicate the direction of significant (*p* < 0.05) age-dependent changes (repeated measures ANOVA factor “age”).

**Figure 5:**
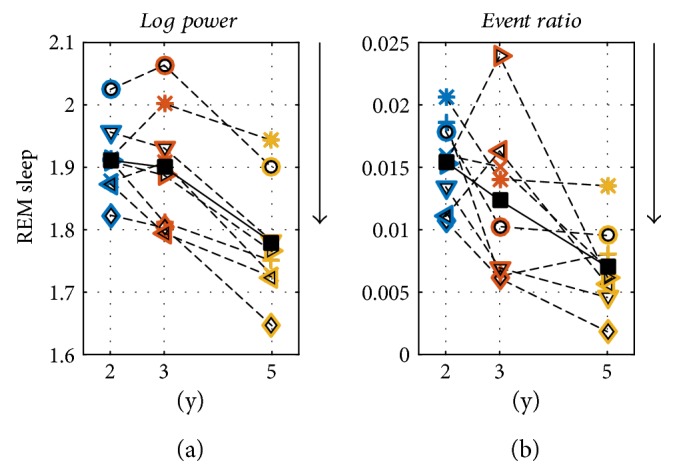
(a) Mean power (log10) and (b) event ratios in the 1.25–5.0 Hz range at three longitudinal time points (2, 3, and 5 years of age) to illustrate the developmental change in REM sleep. For details; see Figure 4.

In a first step, we determined histograms of the events with a frequency bin size of 0.25 Hz (same resolution as for spectral analysis; Figure 1(b)). Next, we computed the event ratio (i.e., ratio between the sum of the durations of the events in this frequency band divided by the total time in the particular stage; Figure 3(b); Figures 4 and 5 mean event ratios of the delta, theta, and sigma bands at three longitudinal time points). The event ratio, combining the duration and the rate of events, is a robust measure of oscillatory activity [14].

**Figure 6:**
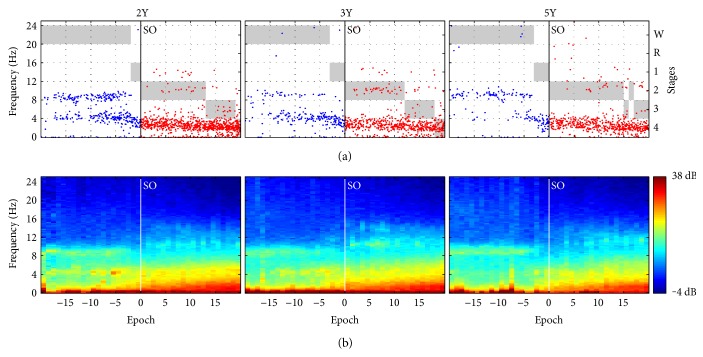
Oscillatory events and spectrograms at the transition into sleep, 10 min prior to and after sleep onset (±20 30 s epochs). Sleep onset was defined as the first occurrence of stage 2. (a) Occurrence of oscillatory events (frequency axis on left) pooled across the eight children superimposed on average sleep stages (axis on right; W: waking; R: REM sleep; 1–4: NREM sleep stages 1–4). (b) Average spectrograms (*n* = 8). Spectra are color coded on a logarithmic scale (0 dB = 1 *μ*V^2^/Hz).

In addition, we investigated whether specific oscillatory events herald the onset of sleep (i.e., the transition from waking to sleep). Sleep onset was operationalized as the first occurrence of stage 2. To track the temporal evolution, data from lights off to sleep onset and the first 10 min of sleep were analyzed (Figure 6, oscillatory events (panel (a)) and mean spectrograms (panel (b)) at the transition into sleep). In the average across subjects, only the 10 min before sleep onset was included.

### 2.4. Further Processing and Statistical Analysis

To investigate developmental trajectories, we examined three specific frequency bands in NREM sleep: delta (1.25–4.0 Hz), theta (4.25–8.0 Hz), and sigma (9.25–13.0 Hz and 13.25–17.0 Hz) and one band (1.25–5.0 Hz) in rapid eye movement (REM) sleep. The bands were determined based on visual assessment of the average power density spectra and the histograms of the event ratios in each subject (Figure 3, mean power density spectra (panel a) and event ratios (panel b); Supplementary Figures S1–S8, corresponding data of individuals). Because of sweating artifacts in two recordings and the sensitivity of the lowest frequency bins to artifacts, we excluded frequency bins up to 1.25 Hz. The longitudinal evolution of events and power in the specific frequency bands was assessed with repeated measures ANOVA (factor “age”). Analyses were performed for light sleep (LightS; NREM sleep stages 1 and 2), slow-wave sleep (SWS; NREM sleep stages 3 and 4), and REM sleep. Delta events started to slow (i.e., a decrease in frequency) before sleep onset (Figure 6(a)); two straight lines were fit through the delta/theta events (2–6 Hz) using their intersection as the start of slowing. The two lines were optimized to best fit the data (i.e., minimal least square error).

## 3. Results

All children slept well as indicated by their average high sleep efficiency [total sleep time as percentage of time in bed; 2Y: 99.2 ± 1.9 (SEM) %; 3Y: 96.4 ± 3.0%; 5Y: 95.9 ± 2.3%]. Average sleep duration was 10.3 ± 0.4 (SEM; 2Y), 10.4 ± 0.8 (3Y), and 10.2 ± 0.4 hours (5Y). The sleep structure of a representative boy at each longitudinal assessment is illustrated in hypnograms (Figure 1).

Oscillatory events detected in the sleep EEG are superimposed on the corresponding spectrogram (Figure 1, Supplementary Figures S1–S8, top panels) for the three longitudinal recordings. Events occurred mainly in delta, theta, and sigma bands. Individual summary data (average power density spectra and event ratio histograms) are illustrated in Supplementary Figures S1–S8 (bottom panels).

Figure 2 exemplifies 10 s epochs of sleep EEG (Figures 2(a), 2(b), 2(c), and 2(d) top) and detected events (rectangles) in combination with color-coded spectrograms (Figures 2(a)–2(d), bottom). Figure 3 illustrates mean power density spectra and event ratios of REM sleep, LightS, and SWS at the three longitudinal time points (corresponding data of individuals are depicted in Figures S1–S8, bottom panels). Figures 4 and 5 depict the longitudinal evolution of mean power and event ratios of the delta, theta, and sigma bands. In Figure 6, oscillatory events (panel (a)) and mean spectrograms (panel (b)) at the transition into sleep (10 min prior to and after sleep onset) are illustrated. In the following, results are described in a frequency and state-specific manner.

### 3.1. Delta Oscillations/Power (1.25–4 Hz) in NREM Sleep

Oscillatory events in the delta range were observed during LightS, SWS, and even in REM sleep (Figures 1 and 3; Supplementary Figures S1–S8). Events occurred most prominently during SWS (Figures 3 and 4) and were paralleled by high power in the corresponding frequency range. The peaks in the mean event ratio around 2 Hz (LightS) and 1.5 Hz (SWS) were not reflected in the mean spectrum. Visual inspection of the whole night spectrogram with the superimposed events (Figure 1 and Figures S1–S8, top) shows the slow waves below 2 Hz in addition to many “fast” delta events forming a band between 2 and 4 Hz in LightS and ranging up to 5 Hz in REM sleep (Figure 3 and Figures S1–S8, bottom). Figure 2 illustrates such events (10 s epochs of sleep EEG and spectrograms with the detected events marked by a rectangle), occurring during REM sleep and LightS. “Fast” delta events were also evident prior to sleep onset (see below; Figure 6). Both power and event ratios in the delta range decreased during the investigated developmental period during LightS and SWS (Figure 4).

### 3.2. Theta Oscillations/Power (4.25–9 Hz) in NREM Sleep

Oscillatory activity in the theta frequency range (bursts of theta activity; Figure 2()) was present most strongly at the beginning of sleep and declined during its nighttime course (Figure 1() and Figures S1–S8). In the mean power spectrum, a peak at 5 Hz was observed in all sleep stages (Figure 3()) that also appeared in the individual power spectra albeit with varying strength and slight variation of frequency (Figures S1–S8, bottom panels). A corresponding peak in the event ratios was observed in LightS only at 2Y and for all ages in SWS. A reduction in theta activity occurred with increasing age (Figures 3() and 4()) for events during both LightS and SWS and for power during LightS only (for REM sleep see below).

### 3.3. Spindles/Sigma Activity (9.25–13 and 13.25–17 Hz) in NREM Sleep

Events in the sigma range were rare, although a clear band of activity in this frequency band was evident in the spectrogram (Figure 1; Supplementary Figures S1–S8). This activity was also reflected by a peak in the power density spectra, particularly during LightS (Figure 3).

The spectrogram clearly indicates the presence of sleep spindles (Figure 2(d)). Generally, due to the larger EEG amplitude compared to adult EEG, sleep spindles are less clearly visible in the raw signal. They became more pronounced at 5Y but were still less clearly evident than in the adult sleep EEG. Classically, a distinction between fast (12–14) and slow (10–12 Hz) spindles is made [18–20]. Two corresponding peaks in the mean power spectrum were observed in only two children (see Supplementary Figures S1 and S5). Therefore, we did not employ this distinction in our analysis.

One child (Figures 1 and S1; subject A) showed “ultrafast” spindle events (≥15 Hz) at 2Y that diminished with increasing age and occurred primarily during SWS. Such “ultrafast” spindles were also observed as isolated events in most other children (see Supplementary Figures). For example, subject F showed a clear peak (≈15 Hz) in spectral power in SWS at 2Y (Figure S6), whereas child D (Figures S4) exhibited fast spindles in the “classical” fast frequency range, which behave more like the “ultrafast” events observed mainly during SWS and diminishing with increasing age.

To differentiate between ultrafast and more classically defined spindle events, we divided the spindle frequency band into “regular”: 9.25–13.0 and “ultrafast”: 13.25–17.0 Hz for statistical analysis. In the “regular” spindle band, we found a significant developmental increase in spectral power during LightS and SWS, while an increase in the spindle event ratio was significant only in LightS. Only a few subjects showed a substantial amount of events in the “ultrafast” range, thus no significant change of the event ratios was observed; however, for those children who exhibited these events, their event ratios decreased with increasing age (Figure 4). The observed increase in spectral power (significant in SWS) can be explained by a spillover from the “regular” spindle peak (see Discussion).

### 3.4. Delta/Theta Oscillations/Activity (1.25–5.0 Hz) in REM Sleep

The occurrence of events in the 1.25–5.0 Hz range during REM sleep is evident as shown in Figures 1 and 3 and Supplementary Figures S1–S8. These events in the high delta/low theta range were most prominent at 2Y and decreased across development (Figure 5(b)). The average event ratio showed a clear peak around 2.5 Hz, while the mean power density spectra exhibited only a small bump at 2.5 Hz but then a more pronounced peak at 5 Hz (Figure 3). Nevertheless, power in this frequency range decreased across age (Figure 5(a)).

### 3.5. Transition to Sleep (Sleep Onset)

We investigated the process of sleep onset (Figure 6). Sleep onset was operationalized as the first occurrence of stage 2; we analyzed data 10 min prior to and after sleep onset (±20 30 s epochs). Oscillatory events were pooled across the eight children; sleep stages were “averaged” (Figure 6(a)) by first determining whether at least half of the children were awake. If yes, wake was assigned as the average stage. If the majority of children were in NREM sleep, the rounded average of the NREM sleep stages (values 1 to 4) was assigned as the average stage. The spectrograms were then averaged (Figure 6(b)).

Average latency to stage 1 was 10.8 ± 2.1 (SEM; 2Y), 13.7 ± 1.3 (3Y), and 14.7 ± 2.9 min (5Y) and to stage 2 (sleep onset) was 12.1 ± 2.2 (SEM; 2Y), 15.7 ± 1.1 (3Y), and 16.4 ± 3.0 min (5Y). No age-dependent differences in sleep latencies were observed.

Alpha events (8–10 Hz) were present throughout waking and disappeared with the onset of stage 1 (Figure 6()). Delta/theta events (2–6 Hz) were observed in waking several minutes before sleep onset. With increasing age, these events appeared closer to sleep onset and slowed shortly before the onset of stage 1. The slowing (see Methods) started 19.8 s (SEM; 2Y), 54.0 s (3Y), and 67.4 s (5Y) or 1-2 epochs before the occurrence of the first epoch of stage 1. After sleep onset, delta events continued to decrease in frequency. Delta (around 4 Hz) and alpha (around 9 Hz) activities were also visible in the spectrograms. Sleep spindles (around 10 and 14 Hz) appeared only about 1 min after sleep onset and were barely visible in the spectrograms (Figure 6()).

## 4. Discussion

In this longitudinal study, we examined developmental changes in oscillatory events and power density spectra of the NREM and REM sleep EEG in children ages 2Y, 3Y, and 5Y. Overall, we observed a decrease of spectral power and event ratios in the delta/theta range and an increase in the sigma frequency range with increasing age. Moreover, we found that specific oscillations disappeared (theta bursts and “ultrafast” spindles) while other ones emerged (“regular” spindles) across the preschool-age years. Similar to adults, large interindividual differences were present thus suggesting that developmental changes in EEG sleep oscillations are not consistently pronounced in all children.

### 4.1. Methodological Aspects

In contrast to standard methods for detecting sleep oscillations, such as those used to identify slow waves (e.g., [21, 22]) or sleep spindles (for a review see [18]), our method does not use predefined frequency bands and is therefore better suited to address large variability in the incidence and frequencies of sleep oscillations across individuals. Moreover, this approach allows observation of “nonstandard” oscillations such as the “ultrafast” spindle events in NREM sleep (Figures 1 and 3) or the delta/theta events in REM sleep (Figure 3). Our event detection provides information complementary to spectral analysis, which is usually applied on longer time scales (here for 30 s segments) compared to 1 s for the event analysis, making it especially sensitive to the variability of the spectral power on shorter time scales. For instance, spectral analysis as an approach does not distinguish between diffuse oscillatory activity and clearly visible temporally localized burst-like oscillations. Additionally, the event analysis relies on a different decomposition of spectral power. Spectral analysis does not provide information about the origin of the spectral power in a specific frequency band, while the event analysis is based on modeling by stochastically driven oscillators or relaxators with each of them producing a more or less broad peak in the power density spectrum. For example, the value of the power in the theta band does not reveal whether this power originates from damped delta oscillations, theta oscillations, or alpha oscillations.

### 4.2. Delta Oscillations/Power in NREM Sleep

Event ratios and power in the delta range during LightS and SWS showed a developmental decline as measured by a reduction in slow waves and “fast” delta oscillations (2–4 Hz). “Fast” delta oscillations were also a marker for the transition into sleep (see discussion below). These findings complement our previous work showing a similar decrease in SWA during afternoon naps in the same early childhood cohort [23] and extend published data revealing that a decline in the level of nighttime SWA continues during adolescence [24–26]. Additionally, our prior results from the same preschool-age sample showed a developmental increase in EEG coherence (a measure reflecting long-range functional connectivity) in the low delta range (0.8–2 Hz) within the left and right hemispheres [10], and in a different sample, we found that coherence of low delta activity increased over adolescence and into early adulthood [11]. Taken together, our current results add to the growing body of longitudinal and cross-sectional data suggesting that the first two decades of life are sensitive developmental windows during which the brain undergoes major reorganization as reflected in not only SWA but also in sleep oscillatory events [24].

“Fast” delta oscillations (>2 Hz) likely have two different generating components, originating in the cortex and/or in the thalamus [9]. Our findings may suggest a specific functional role of “fast” delta oscillations that warrants further investigation.

### 4.3. Delta/Theta Oscillations in REM Sleep

Establishing a clear border between the delta and theta frequency bands is difficult, especially during periods of rapid developmental change in the sleep EEG. In REM sleep, “bands” of oscillatory events with frequencies up to 5 Hz were observed, and fast delta/low theta events were also present in REM sleep and decreased with increasing age. The emergence of this activity was previously reported to occur between 12 and 24 months of age [27]. Such events were also observed with our approach in REM sleep of young adults [12, 13]. An example of a delta event in REM sleep is provided in Figure 2(a). Part of the observed delta/theta oscillations may resemble bursts of sawtooth waves occurring in REM sleep in children [28] and adults [29, 30] or bursts of notched theta activity [28, 31]. Our event density at 2Y was higher than those reported in young adults [29] and approached the magnitude of young adults by 5Y. In young healthy adults, bursts of sawtooth waves are associated with rapid eye movements, and it has been speculated that they may be related to ponto-geniculo-occipital (PGO) spikes originating in the pons [30].

### 4.4. Theta Oscillations/Power in NREM Sleep

Theta oscillations occurred in bursts (Figure 2) in many children in our study. These types of oscillations were not previously observed in adults [12, 13]; thus, we speculate that bursts of theta oscillations are unique to early childhood and diminish during the course of this developmental stage. Additionally, we found a decline of theta activity in NREM SWS sleep that was most pronounced for event density in all children (Figure 4). This finding extends our previously published data indicating a developmental decline of theta power during afternoon and evening naps in the same longitudinal cohort of children [23]. In this analysis, we also observed a developmental decrease in theta power during LightS. Theta activity, in particular at 2Y during LightS, may also be related in part to postarousal hypersynchrony, a distinctive arousal pattern characterized by bursts of 3.0–4.5 Hz with amplitudes of 75 to 350 *μ*V [28].

### 4.5. Spindles/Sigma Activity

In adults, one of the most prominent oscillatory events are sleep spindles, which are observed in the 11–15 Hz frequency range. Many researchers distinguish between slow spindles below 12 Hz that occur more frontally in comparison to those that are observed more centrally with a frequency around 14 Hz [19, 32]. In adults, the frequencies of slow and fast spindles vary between subjects and as a function of sleep cycle and sleep stage [13, 33]. Activity in the spindle frequency range exhibits a U-shaped time course within NREM sleep episodes [33]. In our sample, we detected very few spindles at 2Y and 3Y, although the spindle frequency band was already visible in the spectrogram (Figure 1; Supplementary Figures S1–S8). Both the number of spindles (reflected in the event ratio) and sigma power increased significantly between 2Y and 5Y. In the same cohort, a similar age-dependent increase in sigma power was also observed during naps [23], and coherence in the spindle band showed both an intra- and interhemispheric increase across early childhood [10].

An interesting observation in our subjects was the occurrence of “ultrafast” spindles. Although not present in all children, these oscillatory events exhibited similar properties across subjects, which clearly distinguished them from “normal” spindles in several ways: (i) they occurred in deep sleep with the same or even higher event ratios than in LightS (Figure 4); (ii) the event ratio declined with age; and (iii) the corresponding oscillatory events occurred outside the “normal” sigma frequency band at higher frequencies as shown in Figures S1–S8. For some children, one could even consider these ultrafast spindles as a distinct frequency band (Figure 1). The observed significant developmental increase in spectral power in the 13.25–17.0 Hz range (Figure 4) contradicts an age-dependent decrease of “ultrafast” spindles; however, spectral power is a less local measure in frequency space. Increasing power of the spindle peak(s) between 11 and 13 Hz will also lead to increased spectral power in the neighboring higher frequency bands. Moreover, we cannot exclude that the observed increase in spectral power is due to an unspecific increase of power above approximately 10 Hz (e.g., subject A, Figure S1).

Sleep spindles are a thalamocortical phenomenon. They are generated in the thalamus (reticular nucleus), but the cortical network is involved in their onset and contributes to their termination [34]. Different levels of thalamocortical hyperpolarization may determine spindle frequency, and thalamocortical projections may be responsible for regional differences in spindle characteristics [35]. Furthermore, a recent pharmacological study in humans indicated that different generating mechanisms might underlie fast and slow spindles [36]. How this applies in early childhood remains an open question; however, our results suggest that considerable thalamocortical remodeling/restructuring may occur during early childhood development.

Results from a number of recent studies indicate that spindle characteristics and sigma activity may be markers of cognitive ability. For example, in school-age children, relative sigma power is positively associated with full scale and fluid IQ but not verbal IQ, working memory, and speed of processing [37]. Although less is known about such links in early childhood, our recently published data showed that young children with greater slow sigma power in the parietal region had faster processing speeds [38], and in another study, spindle density during afternoon naps was associated with memory performance in habitually napping preschoolers [39]. Whether the maturational trajectories of spindle characteristics and sigma activity correspond to developing cognitive abilities in early childhood remains an area of rich investigation.

Our present findings extend those from our recently published paper examining early developmental changes in sleep spindle characteristics in the same cohort [15] using a different approach (i.e., band pass filtered EEG with passband corner frequencies of 11–15 Hz and stopband corner frequencies of 10 and 16 Hz). In the present analysis, we employed an automatic algorithm that does not rely on predefined frequency bands. Instead, here, we established the frequency bands based on the frequency distribution of the detected events, leading to a more narrow band between 9.25 and 13.0 Hz for “regular” spindles and a distinct frequency range for the “ultrafast” spindles (13.25–17.0 Hz), which exhibited a decreasing event ratio with age in some children. McClain et al. [15] observed a developmental increase in spindle duration, while spindle density did not show a similar age-related trajectory. In the present analysis, we examined only the event ratio, which combines density and duration. Although the observed increase of the event ratio with age is consistent with the results of McClain et al. [15], visual inspection of Figure 1 and Figures S1–S8 shows that the event density of the “regular” spindles also increases with age. In contrast, McClain et al. [15] reported an almost constant event density across all age groups, meaning that they detected spindles in the younger age groups (2Y and 3Y), which were not detected with the present approach. Furthermore, “ultrafast” spindles were not observed by McClain et al. [15]. These study discrepancies suggest the need for a more detailed analysis at the level of single events.

### 4.6. Transition into Sleep

Sleep onset may be operationalized in various ways. The first epoch other than wakefulness—typically stage 1—is often considered the time of sleep onset [16, 31, 40]. Here, we defined the occurrence of stage 2 as sleep onset [41, 42]. We believe that this approach results in a more reliable boundary between wakefulness and sleep [41, 42] and considers stage 1 as part of the transition into sleep [43].

Our data showed that delta/theta events (2–6 Hz) herald the transition into sleep. These events, however, were present already during waking several minutes before sleep onset, highlighting that the transition into sleep is a gradual process. Delta oscillations started to appear closer to sleep onset with increasing age but still before the onset of stage 1. Furthermore, they began to slow (i.e., became lower in frequency) approximately 2 epochs (50 s) before the first occurrence of stage 1. Alpha events disappeared with the onset of stage 1 sleep, whereas sleep spindles only appeared approximately three epochs after stage 2 was scored. Thus, the slowing of delta events and the disappearance of alpha events most clearly indicated the transition into sleep. This is in line with the observations that drowsiness in children at the age of about 3 years is accompanied by the appearance of diffuse low voltage activity (20–80 *μ*V) intermixed with delta and theta activity [28]. Over 5 decades ago, Gibbs and Gibbs [20] reported a steady slow activity (Figure 37 in [20], most prominent at age 1 year; observed up to 9 years of age) and paroxysmal slow activity (most prominent in 3 to 4 years old) during drowsiness. Taken together, these observations indicate that fast delta events occurring before stage 1 may be a sign of microsleep episodes.

### 4.7. Limitations

Although we used a well-controlled protocol and studied children longitudinally, several limitations should be discussed. First, our study design did not allow napping on the day of overnight sleep recordings during a developmental period in early childhood that is characterized by a transition from a biphasic to a monophasic sleep-wakefulness pattern. Thus, children assessed at younger ages (2Y and 3Y) likely accumulated a higher level of sleep pressure across the waking day than those at age 5Y, a time when none of the participants still napped [44]. The analyzed sleep recordings therefore do not represent true baseline conditions, and the developmental trajectories of delta and spindle activity as well as sleep onset might have been influenced by different levels of sleep pressure. Nonetheless, sleep latencies were not significantly different across the 3 ages; therefore, we assume that sleep pressure did not substantially affect our observed developmental trajectories. Second, our analyses were based on a small homogenous sample of healthy, good-sleeping children, which limits the generalizability of our findings. Third, our analyses were restricted to a central EEG derivation. Based upon recent data, frequency-specific regional aspects of maturational changes exist (e.g., [45, 46]), thus suggesting topographic analysis as a rich approach in future investigations. Finally, one disadvantage of the applied method to detect oscillatory events is that it is not well suited to identify and characterize slow oscillations with frequencies around 1 Hz and below due to the use of segments of 1 s duration; however, using longer segments would deteriorate the temporal resolution of the method, as detailed in [13, 14].

## 5. Conclusion

In this longitudinal study, we found early developmental changes in several oscillatory events in the sleep EEG as well as large interindividual differences in delta, theta, and spindle-like oscillations. Collectively, our data suggest that delta and spindle oscillations reflect maturational changes occurring in the thalamocortical system during early childhood. Whether early changes in oscillatory events are paralleled by children's maturing cognitive capabilities is an area of rich future investigation that may shed light on the underlying neurophysiological substrates and pathways that contribute to variability in the brain and behavioral functioning in the early years of life.

## Supplementary Material

Supplementary Figures S1 to S8: Top: Spectrograms, oscillatory events and hypnograms of individual children (A-H) recorded longitudinally at 2Y (a), 3Y (b) and 5Y (c). For details, see Figure 1. Bottom: Mean power density spectra and event ratios of individual children (A-H) recorded longitudinally. For details, see Figure 3.
