# Supplementary material for: Developmental Changes in Sleep Oscillations during Early Childhood
Source: Neural Plast. 2017 Aug 15;2017:6160959. doi: 10.1155/2017/6160959 (PMC5563422; doi:10.1155/2017/6160959)

Supplementary Material

Developmental changes in sleep oscillations during early childhood

Eckehard Olbrich[1], Thomas Rusterholz[2.3], Monique K. LeBourgeois[4,*], Peter Achermann[2,5,6,7,*]

[1] Max Planck Institute for Mathematics in the Sciences, Leipzig, Germany

[2] Institute of Pharmacology and Toxicology, University of Zurich, Switzerland

[3] University Hospital of Child and Adolescent Psychiatry and Psychotherapy, University of Bern, Bern, Switzerland

[4] Sleep and Development Laboratory, Department of Integrative Physiology, University of Colorado Boulder, Boulder, CO, USA

[5] The KEY Institute for Brain-Mind Research, Department of Psychiatry, Psychotherapy and Psychosomatics, University Hospital of Psychiatry, Zurich, Switzerland

[6] Zurich Center for Interdisciplinary Sleep Research, University of Zurich, Switzerland

[7] Neuroscience Center Zurich, University and ETH Zurich, Switzerland

[*] shared last authorship

Corresponding Author:

Eckehard Olbrich, PhD

Max Planck Institute for Mathematics in the Sciences

Inselstraße 22, 04103

Leipzig, Germany

e-mail: olbrich@mis.mpg.de

Tel. +49 341 9959 568 | Fax +49 341 9959 658

Supplementary Figures S1 to S8: ***Top:*** Spectrograms, oscillatory events and hypnograms of individual children (A-H) recorded longitudinally at 2Y (a), 3Y (b) and 5Y (c). For details, see Figure 1. ***Bottom:*** Mean power density spectra and event ratios of individual children (A-H) recorded longitudinally. For details, see Figure 3.

Figure S1: CHILD A

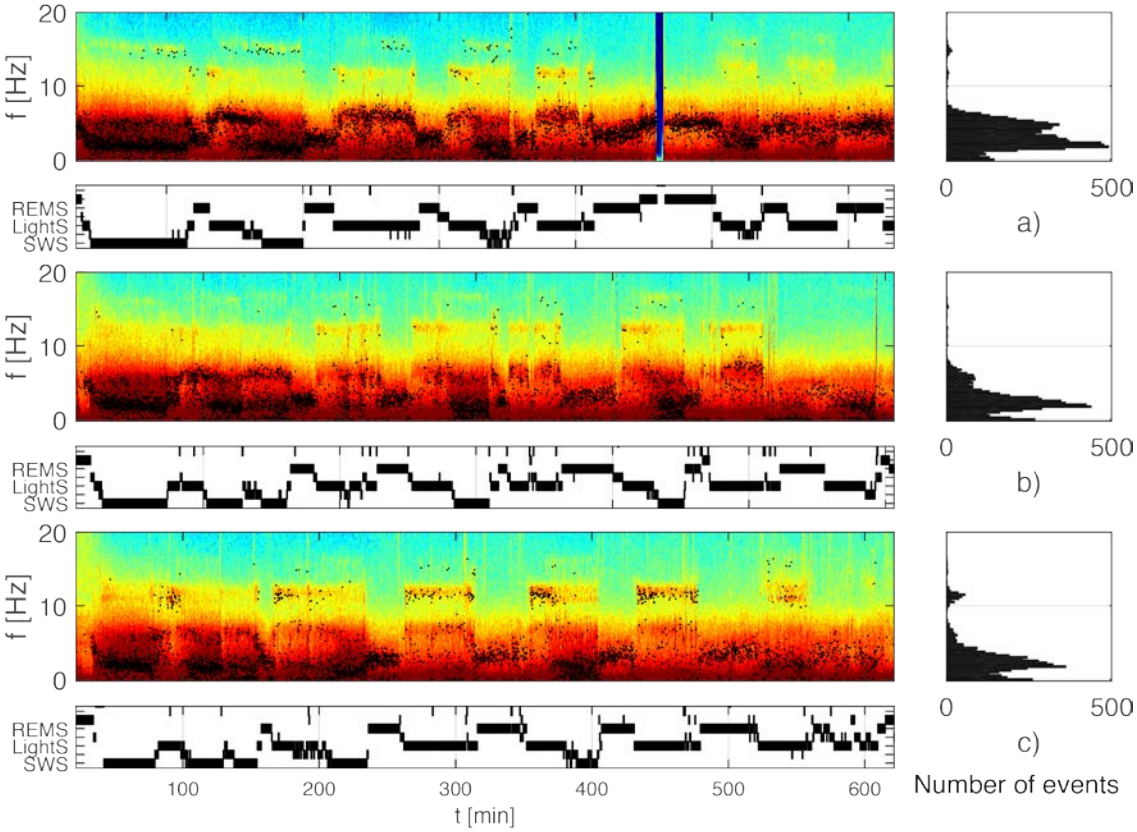

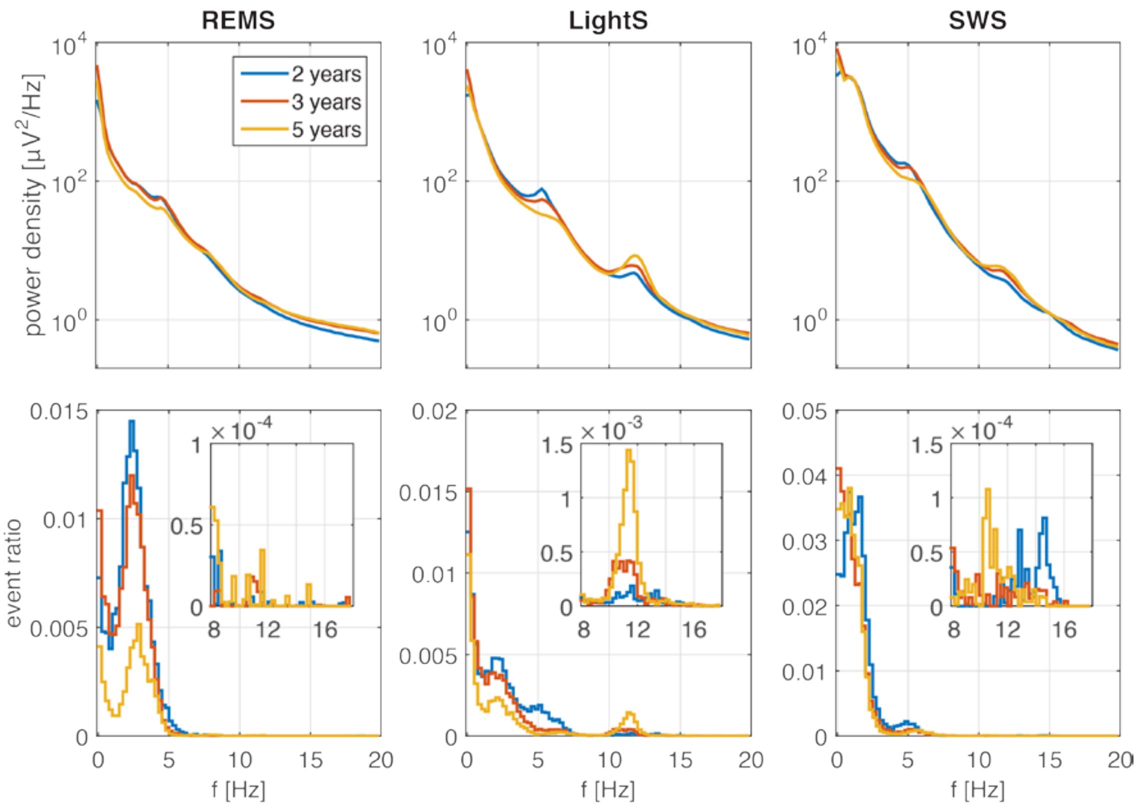

Figure S2: CHILD B

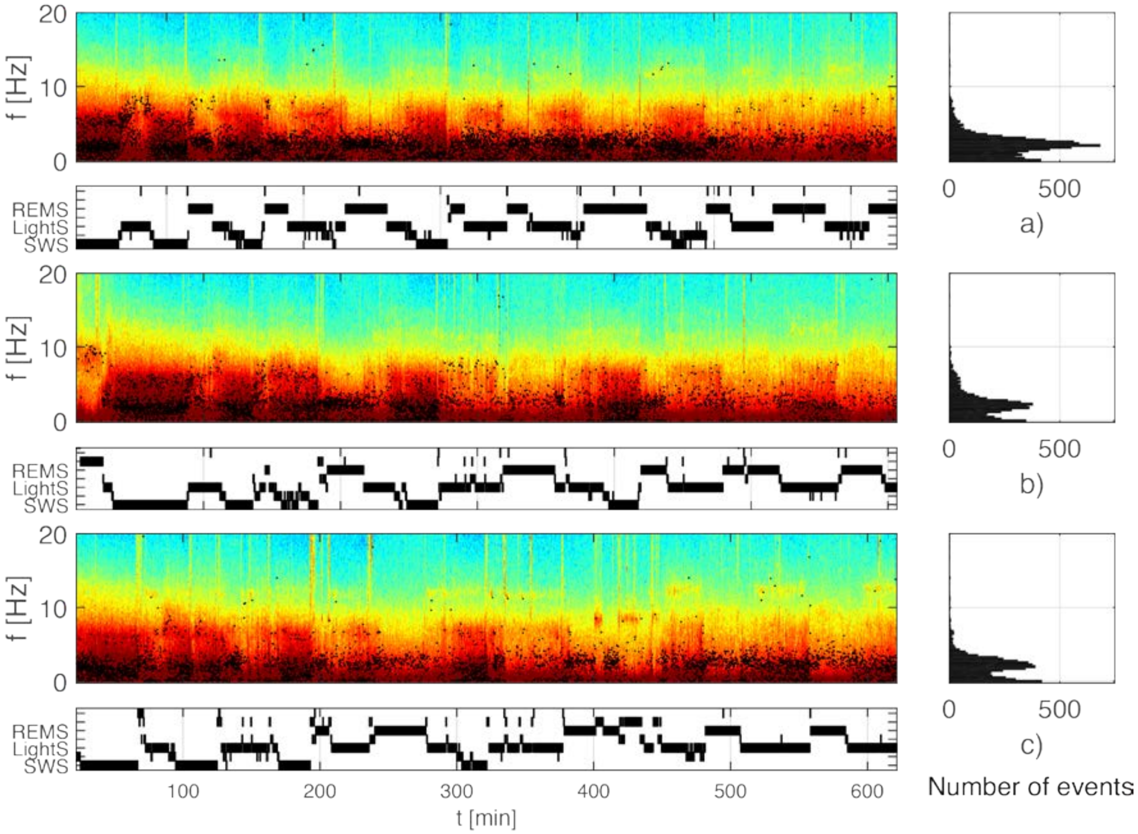

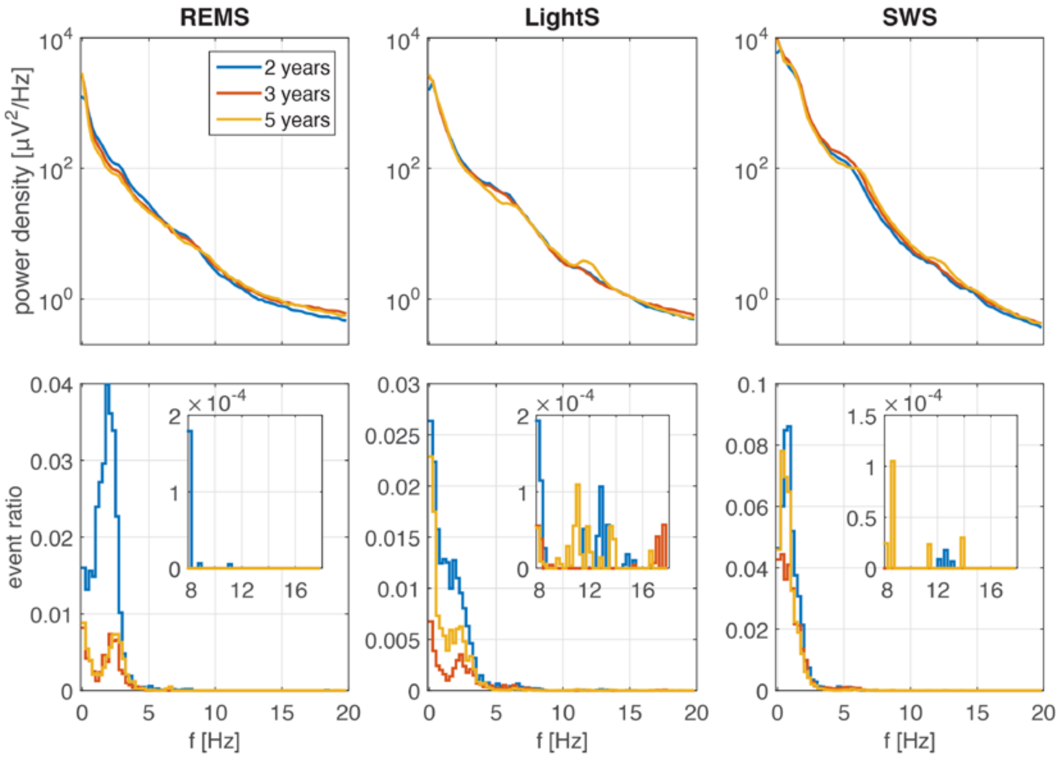

Figure S3: CHILD C

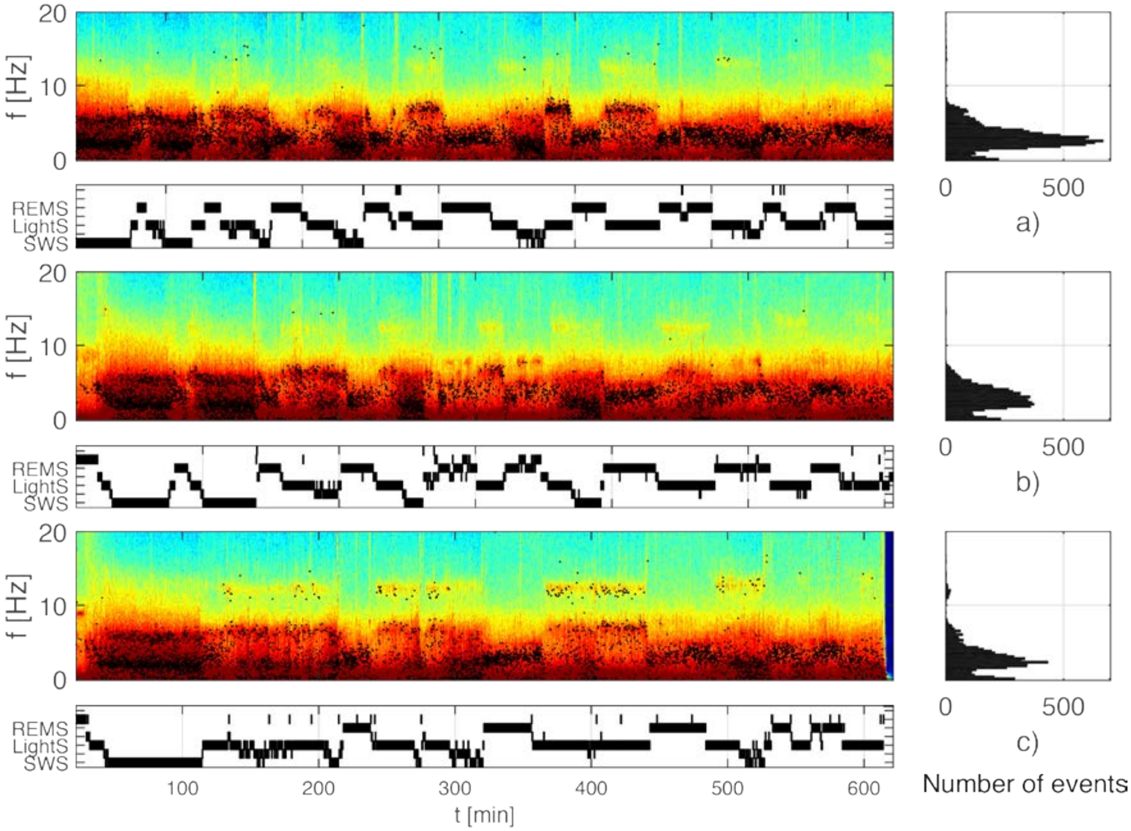

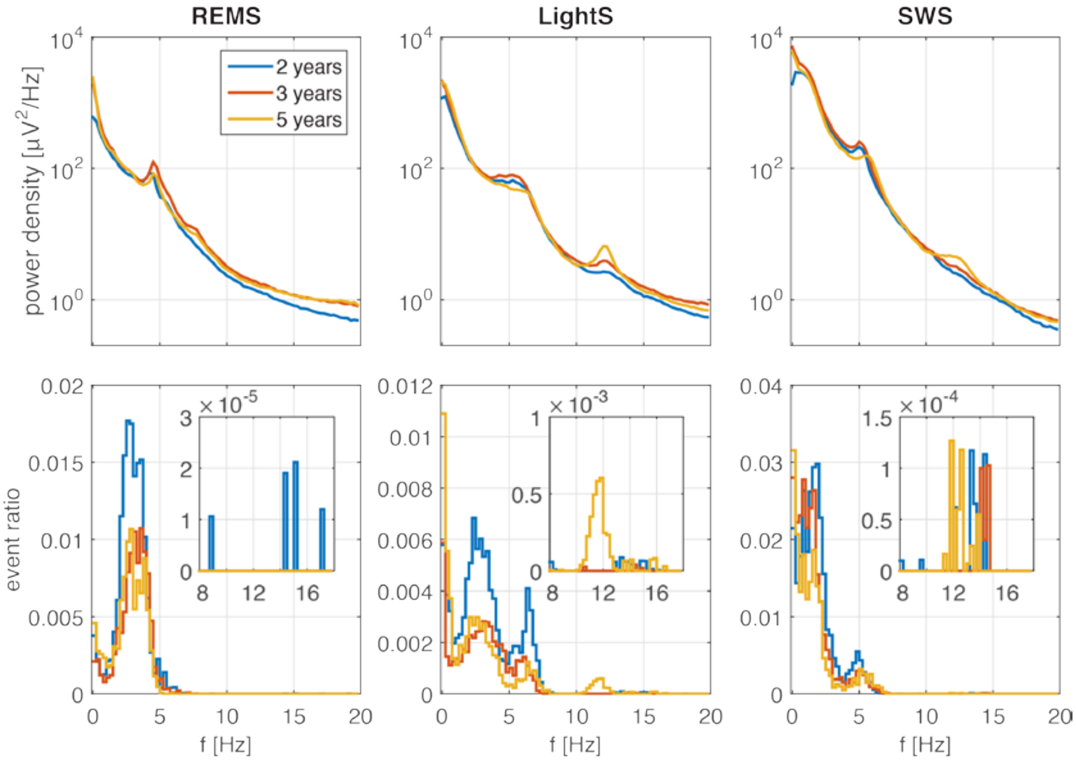

Figure S4: CHILD D

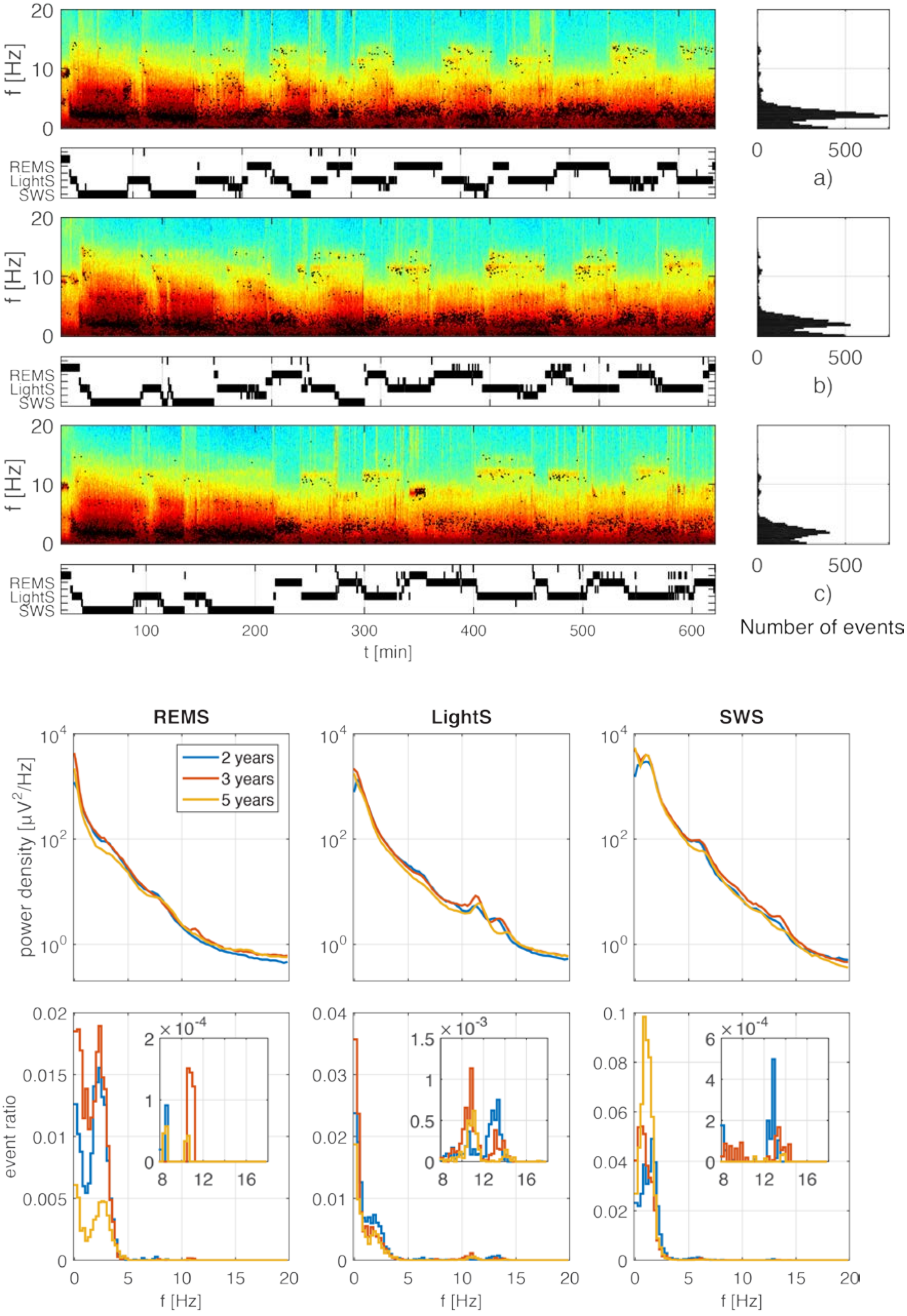

Figure S5: CHILD E

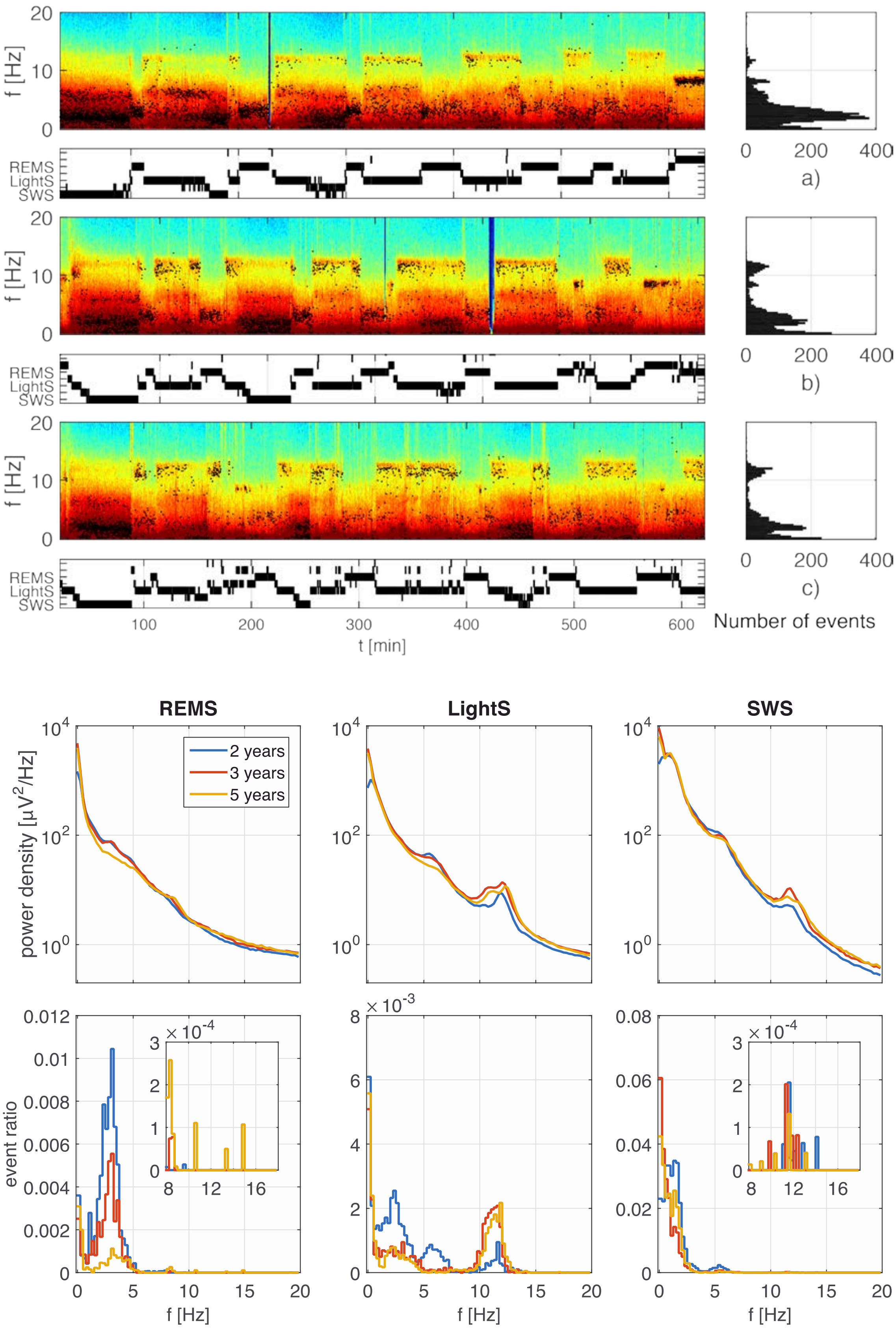

Figure S6: CHILD F

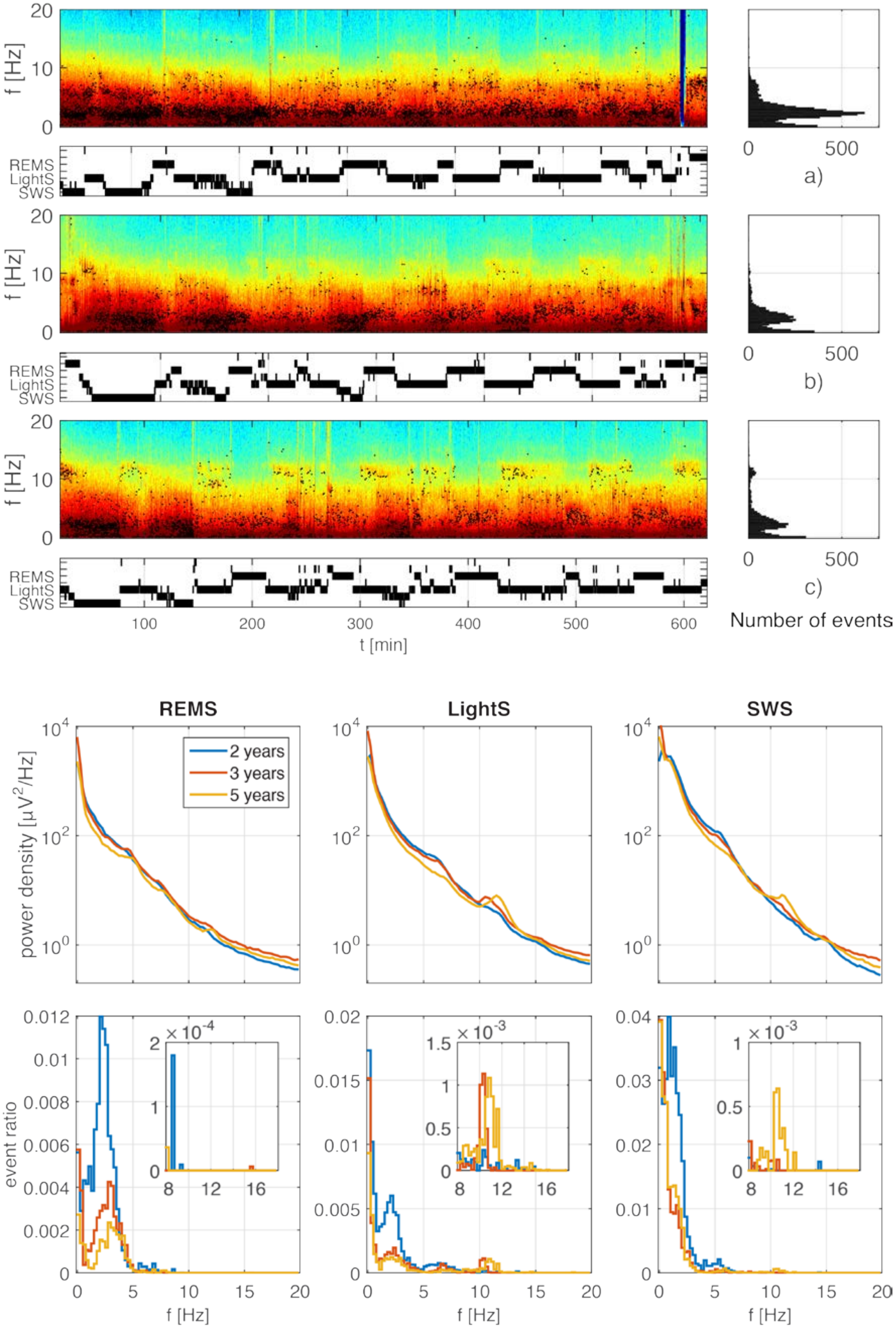

Figure S7: **CHILD G**

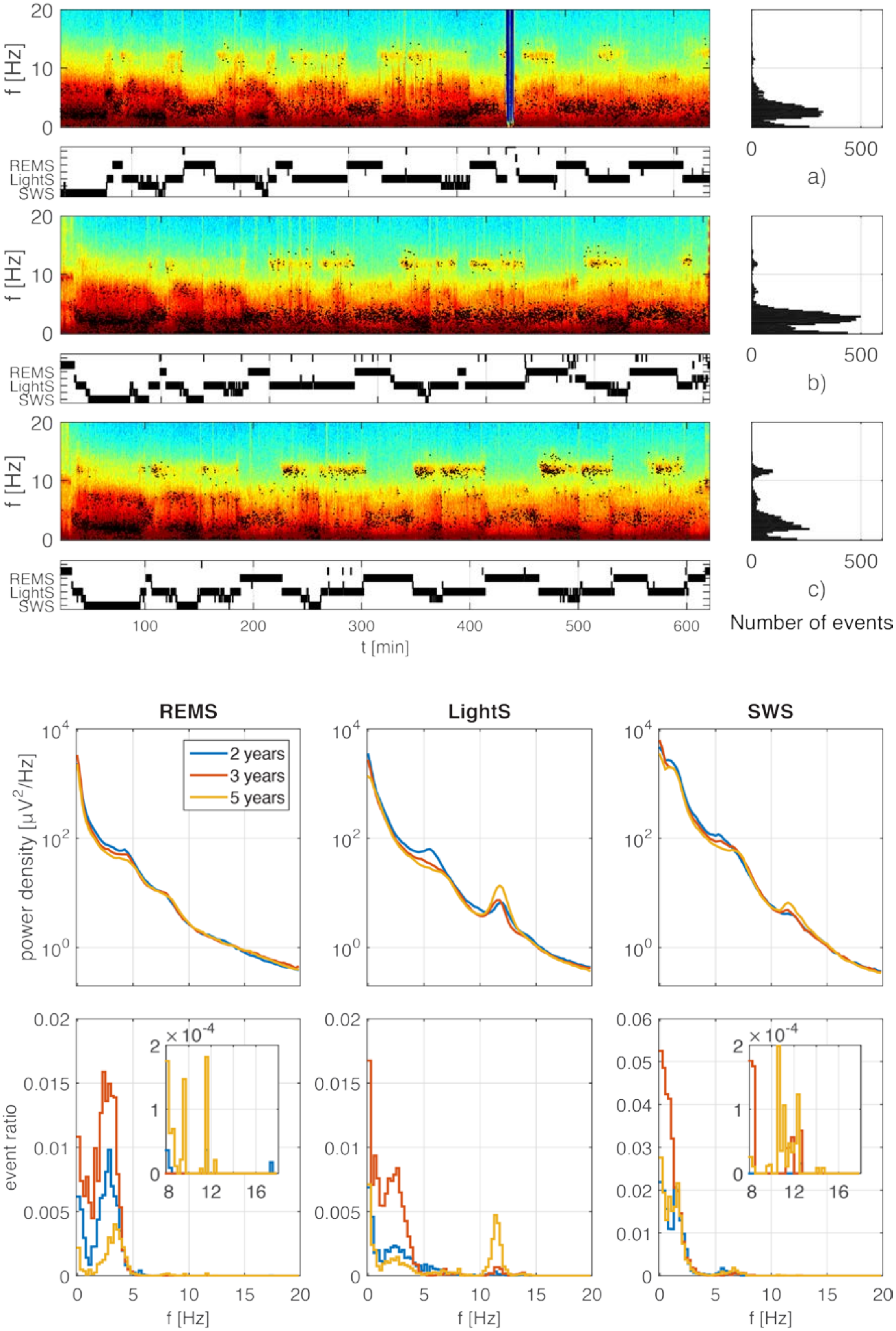

Figure S8: **CHILD H**

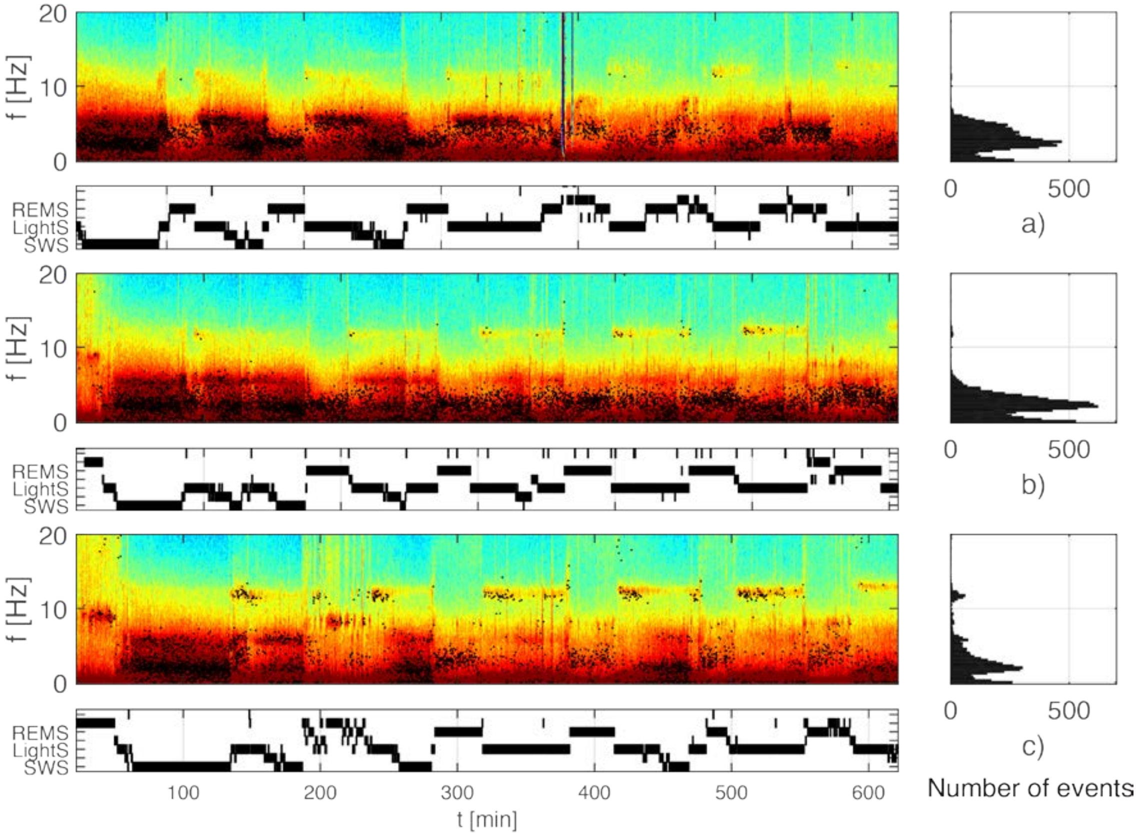

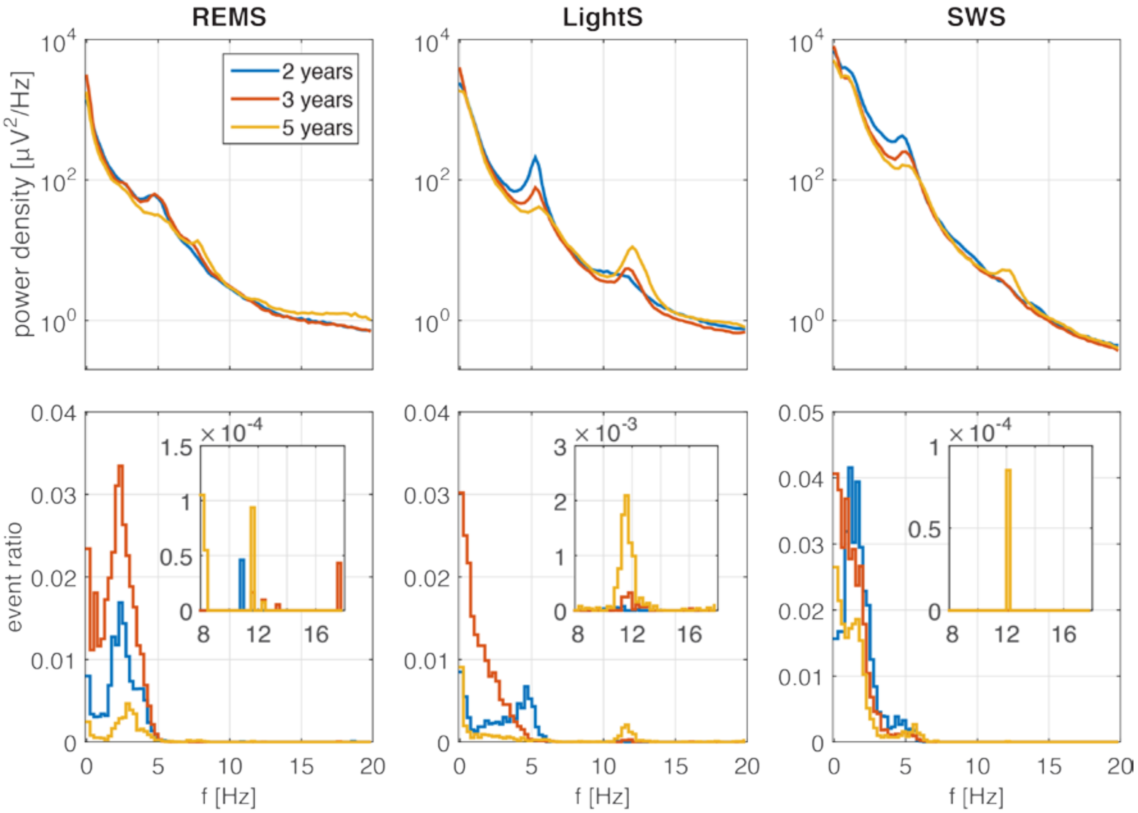

Supplement: Supplementary file 1 — Supplementary Figures S1 to S8: Top: Spectrograms, oscillatory events and hypnograms of individual children (A-H) recorded longitudinally at 2Y (a), 3Y (b) and 5Y (c). For details, see Figure 1. Bottom: Mean power density spectra and event ratios of individual children (A-H) recorded longitudinally. For details, see Figure 3. [file 6160959.f1.pdf]
